# Diagnosis of Cervical Spinal Cord Multiple Sclerosis by a Chiropractic Physician: A Case Report

**DOI:** 10.7759/cureus.62618

**Published:** 2024-06-18

**Authors:** Steven P Brown

**Affiliations:** 1 Integrative/Complementary Medicine, Brown Chiropractic & Acupuncture, PC, Gilbert, USA

**Keywords:** chiropractic, history, diagnosis, imaging, multiple sclerosis

## Abstract

We present a case report of diagnosis of cervical spine multiple sclerosis by a chiropractic physician. This unique case contributes an account of a challenging differential diagnosis to the literature. A 30-year-old male presented with a three-year history of diffuse left upper extremity motor strength deficits and paresthesia (numbness and tingling). The patient had seen multiple physicians for these symptoms with no diagnosis of multiple sclerosis and no advanced imaging. The differential diagnosis included lower cervical spine nerve root compression or neurological disorders such as amyotrophic lateral sclerosis, cerebral lesion, motor neuropathy, multiple sclerosis, or spinal cord lesion. MRI of the cervical spine with and without IV contrast revealed evidence of spinal cord multiple sclerosis. The patient was referred to a neurologist where the diagnosis of multiple sclerosis was confirmed. A 10-year follow-up showed the patient was controlling his condition with medications and had no disability. This case underscores the importance for physicians to consider neurological conditions and advanced imaging in the presence of diffuse motor strength deficits and paresthesia in the absence of injury, pain, or any other symptoms.

## Introduction

A case report of diagnosis of spinal cord multiple sclerosis (MS) by a chiropractic physician is rare. A literature search revealed no other case reports of this nature. Two case reports of diagnosis of brain MS by a chiropractic physician were found [1,2].

MS is a chronic autoimmune disease of the central nervous system (CNS) characterized by inflammation, demyelination, gliosis, and neuronal loss. MS can affect the brain and/or the spinal cord. Pathologically, perivascular lymphocytic infiltrates and macrophages produce degradation of myelin sheaths that surround neurons. Neurological symptoms vary and can include vision impairment, paresthesia (numbness and tingling), focal weakness, bladder and bowel incontinence, and cognitive dysfunction. Symptoms vary depending on lesion location. Lesions in the CNS occur at different times and in different CNS locations. Because of this, multiple sclerosis lesions are sometimes said to be "disseminated in time and space". Clinical symptoms characterized by acute relapses typically first develop in young adults [3].

The diagnosis of MS can be challenging. The physician often faces a nonspecific and/or atypical clinical presentation. This may result in diagnostic confusion and delayed treatment. We present this case report to highlight the need for timely and accurate diagnosis of MS. By using disease-modifying agents, a reduction in the frequency and severity of relapses as well as a decrease in brain lesion development can occur [2]. Physicians should consider neurological disorders and advanced imaging in the presence of diffuse motor strength loss and paresthesia in the absence of trauma, pain, or any other symptoms.

## Case presentation

Subjective examination

**Figure 1:**
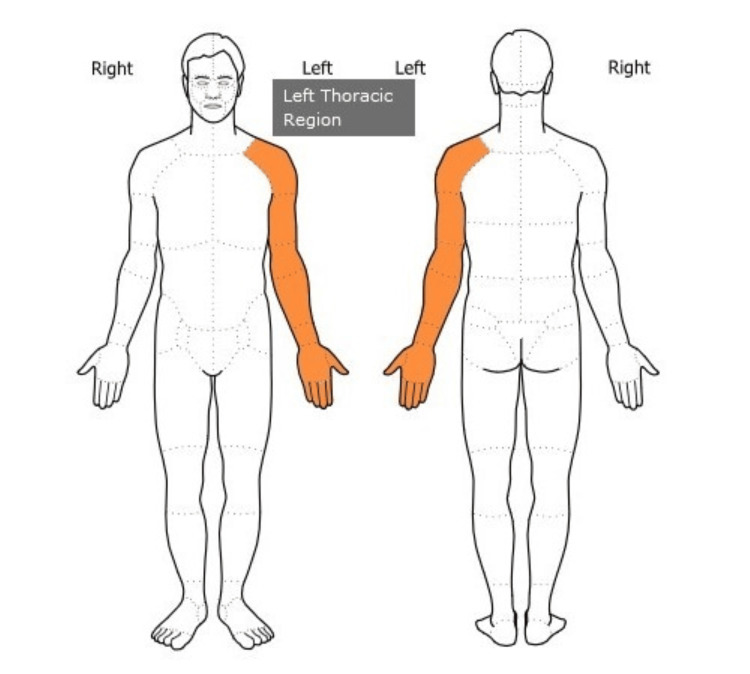
Symptom Diagram Area of left upper extremity motor strength loss and paresthesia (numbness and tingling) symptoms reported by the patient. Image created by the author.

In October 2014, an otherwise healthy 30-year-old white male police officer presented to our chiropractic office with a chief complaint of left upper extremity motor strength loss and paresthesia (numbness and tingling) which had been present on and off for three years since 2011 (Figure 1). The symptoms started in the left upper extremity three years ago, went to the right upper extremity for a while, and returned to the left upper extremity. These symptoms had been constant and worse for the past two months. The patient did not report neck pain, left arm pain, headache, or any other symptoms. The patient’s health history was unremarkable and was negative for trauma, medication use, relevant occupational hazards, co-morbidities, autoimmune disease, or neurological disorders.

Timeline of prior treatment

Medical records of prior treatment were not available for review. This is the timeline of prior treatment as reported by the patient at the time of initial consultation in 2014.

The patient reported seeing a pain management specialist for these symptoms three years earlier in 2011. This physician performed EMG testing of the upper extremity which was normal. They did not order any cervical spine imaging. The patient reported that the provider performed some type of cortisone injection into the neck and shoulder regions. The patient reported that this treatment gave six to seven months of relief and then symptoms returned.

The patient reported seeing a chiropractic physician for these symptoms earlier in 2014 who performed cervical spine radiographs and manual cervical spine manipulation with no improvement in symptoms.

The patient reported seeing an acupuncturist for these symptoms and having one acupuncture treatment with no improvement in symptoms.

The patient reported that he was currently seeing another chiropractic physician for these symptoms and had seen that provider four to five times. This provider performed cervical spine decompression and deep tissue massage therapy with no improvement in symptoms. This physician did not order any cervical spine imaging.

The patient reported he had not had any physical therapy for this condition. The patient had been dealing with these symptoms for three years and was referred to our office by a colleague at work.

Objective examination

Vital signs were within normal limits. Cervical spine active and passive range of motion testing performed visually was within normal limits without pain.

Reflex examination showed biceps, triceps, and brachioradialis deep tendon reflexes were normal bilaterally, graded +2 (Table 1). A limitation of our reflex evaluation was that we did not include upper motor neuron reflexes such as the Babinski reflex or the Hoffman reflex. Upper motor neuron reflex testing may have provided evidence of spinal cord pathology. C5, C6, C7, C8, and T1 dermatome testing was normal bilaterally, graded +2 (Table 2).

**Table 1:** Results of reflex examination

Deep Tendon Reflex	Right	Left	Deep Tendon Reflex Grading Scale
C5: Bicep	+2	+2	0 No response
C6: Brachioradialis	+2	+2	+1 Diminished/low normal
C7: Triceps	+2	+2	+2 Normal
			+3 Increased/brisk
			+4 Very brisk or clonus

**Table 2:** Results of dermatome testing

Dermatome	Right	Left	Sensory Grading Scale
C5: Deltoid	+2	+2	0 No sensation
C6: Anterior arm	+2	+2	+1 Hypoesthesia
C7: Lateral arm & forearm	+2	+2	+2 Normal sensation
C8: Medial arm & forearm	+2	+2	+3 Hyperesthesia
T1: Medial forearm	+2	+2	

Diffuse weakness in the left upper extremity was noted on myotome testing. C5 shoulder abduction, C6 wrist extension, C7 wrist flexion, C8 finger flexion, and T1 finger abduction muscle strength testing was graded 5/5 on the right. However, the same testing was graded 4/5 on the left (Table 3).

**Table 3:** Results of myotome testing

Myotome	Right	Left	Medical Research Council Manual Muscle Testing Scale
C5: Shoulder abduction	5/5	4/5	0 None: No visible or palpable contraction
C6: Wrist extension	5/5	4/5	1 Trace: Visible or palpable contraction (only slight)
C7: Wrist flexion	5/5	4/5	2 Poor: Full range of motion with gravity eliminated
C8: Finger flexion	5/5	4/5	3 Fair: Full range of motion against gravity
T1: Finger abduction	5/5	4/5	4 Good: Full range of motion against gravity w/moderate resistance
			5 Normal: Full range of motion against gravity w/maximum resistance

Orthopedic testing of the cervical spine was unremarkable. Testing consisted of Cervical Distraction Test, Jackson Compression, Maximum Cervical Compression, Shoulder Depression, Soto Hall, and Spurling’s Test which were all negative bilaterally. Notably, there was no positive orthopedic testing for left cervical spine nerve root compression that would explain the patient’s left upper extremity motor strength loss and paresthesia.

Orthopedic testing of the shoulders consisted of Apley’s Superior/Inferior Scratch Test, Dawbarn’s Sign, and Supraspinatus Tests which were negative bilaterally. The left Suprapinatus Test elicited 4/5 weakness but no pain.

Assessment

The differential diagnosis included compressive neuropathy of the left cervical spine nerve roots or neurological disorders such as amyotrophic lateral sclerosis, cerebral lesion, motor neuropathy, MS or spinal cord lesion [4]. There were no objective findings of a left cervical spine nerve root compressive neuropathy, and the broad distribution of weakness in the left upper extremity was not characteristic of cervical spine radiculopathy. A neurological disorder could not be ruled out; therefore, a neurological disorder was given priority in the differential diagnosis.

Plan

No treatment was performed as there was no definitive diagnosis. Our office ordered a cervical spine 3 Tesla (3T) magnetic resonance imaging (MRI) examination with and without intravenous (IV) contrast.

As we suspected MS, MRI examination with IV contrast agent was indicated. MRI with IV contrast is used to evaluate patients for headache, seizures, demyelinating disease, and suspected mass. Post-contrast-enhancing lesions indicate active disease in a patient undergoing imaging evaluation for MS [5].

Imaging

Cervical spine 3T MRI examination with and without IV contrast revealed no evidence of disc protrusion or disc extrusion. There was no spinal stenosis. The neural foramina demonstrated no abnormality.

**Figure 2:**
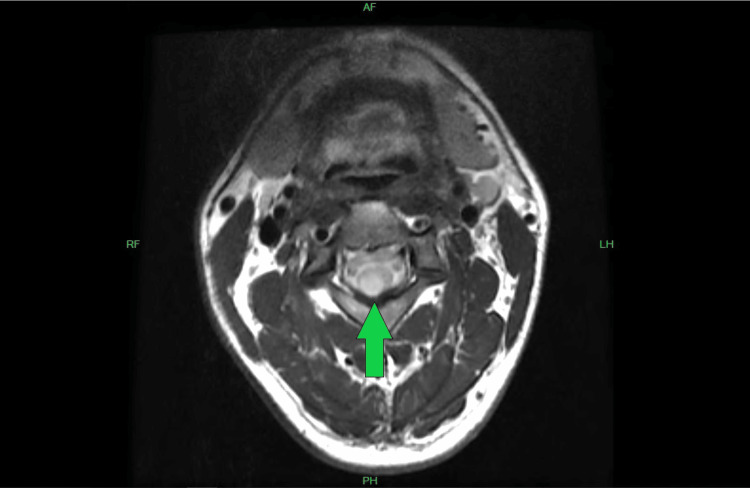
Cervical Spine 3 Tesla MRI Axial View Without Intravenous Contrast There was an abnormal signal noted within the cervical spinal cord from the C2-C3 level to the top of the C4 level spanning 24 mm in height, 8.5 mm in transverse dimension, and 7.7 mm in anterior/posterior dimension. This had the appearance of expanding past the posterior right aspect of the spinal cord (green arrow).

There was an abnormal signal noted within the cervical spinal cord from the C2-C3 level to the top of the C4 level spanning 24 mm in height, 8.5 mm in transverse dimension, and 7.7 mm in anterior/posterior dimension. This had the appearance of expanding past the posterior right aspect of the spinal cord (Figure 2).

**Figure 3:**
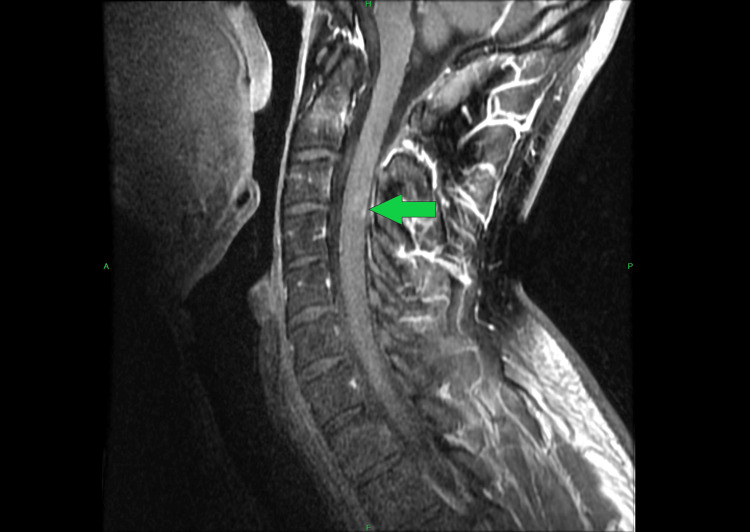
Cervical Spine 3 Tesla MRI Sagittal View With Intravenous Contrast On the sagittal postcontrast images, there was suggestion of minimal specks of enhancement noted at the more inferior aspect of the lesion (green arrow).

Following IV contrast administration there was no evidence of enhancement on the axial images. However, on the sagittal postcontrast images, there was suggestion of minimal specks of enhancement noted at the more inferior aspect of the lesion. Main considerations noted by the radiologist were whether this was sequelae of demyelination or dysmyelination versus a low-grade astrocytoma (Figure 3).

Referral

We discussed the MRI findings with the radiologist and referred the patient to a neurologist for further evaluation and treatment. The neurologist confirmed a diagnosis of multiple sclerosis. The neurologist ordered brain MRI imaging with no demyelinating changes noted in the brain in 2014. The patient continued to have their MS managed by the neurologist. The patient required no further evaluation or treatment in our chiropractic office.

Follow-up imaging studies and medical care

Not all follow-up imaging studies and medical records were available for review. No follow-up cervical spine MRI examinations were available for review. Brain MRI examinations that were available for review are summarized in Table 4.

**Table 4:** Available follow-up brain MRI imaging

Year	Imaging Study	Relevant Radiological Findings
2018	3 Tesla Brain MRI with and without intravenous contrast	Stable demyelinating plaques. No active demyelination.
2019	3 Tesla Brain MRI with and without intravenous contrast	Stable demyelinating plaques. No active demyelination.
2020	3 Tesla Brain MRI with and without intravenous contrast	Stable demyelinating plaques. No active demyelination.
2023	3 Tesla Brain MRI with and without intravenous contrast	Stable demyelinating plaques. No active demyelination.

At his 2023 neurology follow-up, the patient’s MS symptoms were bilateral hand numbness and severe fatigue. The hand numbness was managed with the use of glatiramer for the past eight years. The fatigue was managed with Adderall and Nuvigil. 2023 3T brain MRI with and without IV contrast showed stable demyelinating plaques with no evidence of active demyelination.

MS normally follows a gradually progressive course with permanent disability in 10 to 15 years [3]. In 2024, 10 years after diagnosis, and 13 years after symptom onset, this patient’s MS was stable and managed with medications. He still works as a police officer.

## Discussion

In this case, we present an account of a challenging diagnosis of a neurological condition, multiple sclerosis. This was an atypical presentation of diffuse unilateral upper extremity motor deficits and paresthesia with no other significant clinical findings or red flags. This nonspecific presentation excluded many common musculoskeletal conditions. In the absence of injury, pain, stiffness, restricted range of motion, or positive orthopedic testing, no chiropractic treatment was indicated. Further diagnosis with advanced imaging was indicated.

Interprofessional communication and cooperation between the chiropractic physician, the radiologist and the neurologist facilitated prompt confirmation of the diagnosis. This case illustrates that chiropractic physicians can play an important role in the diagnosis of neurological disorders.

One of the most telling and atypical symptoms reported by the patient was left upper extremity motor strength deficits and paresthesia switching from the left upper extremity to the right and back again. Symptoms of left cervical spine nerve root compression would not be likely to switch to the right and back. However, MS lesions in the CNS can occur at different times and in different CNS locations [3].

Clinical decision-making

A key strength of the clinical approach taken by our chiropractic office was ordering cervical spine MRI without performing any treatment. Amorin-Woods and Parkin-Smith proposed a three-question model of clinical decision-making for chiropractic physicians (Table 5) [6]. Applying these three questions to the current case we find that our office did not have enough information to answer the first two questions.

**Table 5:** The Three-Questions Model of Clinical Decision-Making for Chiropractors

The Three-Questions Model of Clinical Decision-Making for Chiropractors
1. What is the likelihood that I will delay access to more appropriate care for this patient?
2. Is my proposed treatment safe? (What is the likelihood of making this patient worse?)
3. Do I have enough information to answer the first two questions?

As we could not reach a definitive diagnosis, it was possible that chiropractic treatment could have delayed access to more appropriate care for this patient. Further, without a definitive diagnosis, it could not be determined if chiropractic treatment would have made the patient worse. Therefore, the clinically indicated course of decision-making was to order advanced imaging to further the diagnostic process.

## Conclusions

This is a unique case of diagnosis of cervical spinal cord MS by a chiropractic physician. This case provides two crucial takeaway insights. First, with a presentation of diffuse motor strength deficits and paresthesia in the absence of trauma, neck pain or any other symptoms, physicians should consider neurological disorders and advanced imaging. Second, chiropractic physicians should consider that not all patients present with conditions that require chiropractic treatment.
